# Glycolytic Metabolism Is Critical for the Innate Antibacterial Defense in Acute *Streptococcus pneumoniae* Otitis Media

**DOI:** 10.3389/fimmu.2021.624775

**Published:** 2021-04-19

**Authors:** Fangmei Fan, Yurong Ma, Rongshuang Ai, Zhiqiang Ding, Dingyi Li, Yiting Zhu, Qian He, Xinxin Zhang, Yilin Dong, Yujuan He

**Affiliations:** ^1^ Department of Laboratory Medicine, Key Laboratory of Diagnostic Medicine (Ministry of Education), Chongqing Medical University, Chongqing, China; ^2^ Department of Laboratory Medicine, Guiyang Maternity & Child Health Hospital, Guiyang, China; ^3^ Department of Laboratory Medicine, The Second Affiliated Hospital of Kunming Medical University, Kunming, China; ^4^ School of Computer Science, Chongqing Institute of Engineering, Chongqing, China

**Keywords:** glycolysis, otitis media, innate immunity, neutrophils, hypoxia inducible factor-1α

## Abstract

**Objective:**

*Streptococcus pneumoniae* (*S.pn*) is a common respiratory pathogen and a frequent cause of acute otitis media (AOM) in children. However, little is known about the immunometabolism during AOM. This study was to assess the presence of glucose metabolic reprogramming during AOM and its underlying mechanism affecting inflammatory response and middle ear injury.

**Methods:**

The levels of glycolytic metabolism were evaluated by measuring the expression of glycolysis-related genes and the production of metabolites. HE stain, immunofluorescence, immunohistochemistry, enzyme-linked immunosorbent assay (ELISA) and Western blot were performed to measure the effect of glucose metabolic reprogramming on inflammatory response, pneumococcal clearance, hypoxia-inducible factor 1 alpha (HIF-1α) expression and cytokine secretion during AOM, respectively.

**Results:**

The analysis of microarray revealed an increase of the expression of glycolysis-related genes during *S.pn*–induced AOM, which was verified by real-time PCR. Increased glycolysis promoted the production of IL-1β and TNF-α and facilitated the clearance of *S.pn* by enhancing phagocytosis and killing capability of neutrophils, but also aggravated the middle ear injury. Furthermore, these pathogenic effects could be reversed after glycolytic inhibitor 2DG treatment. Additionally, HIF-1α was observed to involve in glycolytic metabolism during AOM.

**Conclusion:**

*S.pn* infection induced increased glycolysis conversion during AOM, which promoted inflammatory responses and bacterial clearance, but also aggravated tissue damage.

## Introduction

Acute Otitis Media (AOM) is one of the most prevalent infectious diseases worldwide, especially in children (1). It has been shown that bacteria can be isolated from middle ear lavage fluid (MELF) of about 70% AOM patients, and 50% of which are *S.pn* (2). Currently, antibiotic usage is the major treatment for *S.pn*-induced AOM (3). However, the emergence of multiple resistant *S.pn* has seriously diminished the outcome of antibiotic treatment of AOM (1). Therefore, it is urgent to explore alternative therapies based on new insights into the pathophysiology of AOM induced by *S.pn*.

Recently, immunometabolism has become an exciting new field with the potential of being novel therapeutic targets for infection and autoimmune diseases (4). In 1923, Otto Warburg, a German biochemist, proposed that tumor cells rely on glycolysis to provide energy even in oxygen rich conditions. Accumulating studies have found that not only tumor cells but also activated immune cells, including macrophages, dendritic cells, Th17 cells and so on, have undergone “metabolic reprogramming” (5–9). As far as glucose metabolism is concerned, it was found that in macrophages and dendritic cells, Toll-like receptor (TLR) activation results in oxidative phosphorylation shifts to glycolysis (7, 10, 11). Furthermore, emerging evidence has shown that metabolic reprogramming in stimulated host immune cells was implicated in the regulation of innate immune function, such as phagocytosis, secretion of pro-inflammatory cytokines, recruitment of immune cells, and production of reactive oxygen species (ROS) (12–17). Our previous studies have demonstrated that TLR2/4 activation plays crucial roles in the immune responses during AOM. Besides, neutrophils are the most predominant innate defenders against *S.pn*, which rapidly migrate to middle ear cavity upon infection (18, 19). Moreover, it is well known that the mucous epithelium of middle ear undergoes extensive modification of immune response during OM, including the recruitment of nonresident leukocyte species (20). However, no study has investigated the features of metabolic changes of middle ear epithelial cells and neutrophils recruited to the middle ear cavity. Given that metabolic changes have proved essential for some key immune-regulatory events downstream of TLR activation, we hypothesized that metabolic reprogramming involved in the innate immune responses during AOM.

The hypoxia inducible transcription factor-1(HIF-1) is a heterodimer composed of two subunits, HIF-1α and HIF-1β, in which HIF-1β subunit is constitutively expressed, while HIF-1α is a transcription-induced nuclear protein. It is known that HIF-1 has nearly 100 target genes involved in hypoxia adaptation, inflammation development, and tumor growth, such as VEGF, EPO, GLUT1, LDHA, PKM2, PDK1, IL-1β, etc. (17, 21–24). Furthermore, HIF-1 is widely expressed in immune cell populations including macrophages, neutrophils, dendritic cells, as well as in T and B lymphocytes and immune lymphoid cells (24–26). A large number of studies have shown that HIF-1α regulated the conversion of energy metabolism to glycolysis and then regulated the functions of myeloid cells, including aggregation, migration, invasion and bacterial killing (16, 24, 25, 27–30). The absence of HIF-1α in neutrophils inhibited ATP generation and reduced their ability to kill bacteria, making mice more susceptible to bacterial infections (26, 28, 31, 32). However, it remains unclear whether HIF-1α participates in metabolic changes and inflammatory defense during AOM.

In our study, we investigated the features and roles of cellular metabolic changes in a mouse model of AOM triggered by transbullar injection with *S.pn*. In detail, we examined the regulation of *S.pn* on the glycolytic metabolism of middle ear epithelial cells and neutrophils recruited into the middle ear, which represent non-immune cell types and innate immune cell types respectively. Our findings suggest that HIF-1α participates in glycolysis metabolism during AOM and the increased glycolysis promotes inflammatory responses, facilitates *S.pn* clearance and aggravates middle ear injury.

## Materials and Methods

### Mice

C57BL/6 mice aged 6 to 8 weeks were obtained from Chongqing Medical University. All mice were housed under a specific-pathogen-free environment in the laboratory animal center of Chongqing Medical University. All procedures involving animals were performed in accordance with the Institutional Animal Care and Use Committee’s guidelines at Chongqing Medical University.

### Reagents

2-Deoxy-d-glucose (2DG#D8375,glycolysis inhibitor) was obtained from Sigma (St. Louis, MO, USA). Mouse anti-HIF-1α antibody was obtained from Abcam (#ab16066, Cambridge, MA, USA).

### Bacterial Strain

Streptococcus pneumonia clinical isolate 31693 (serotype 19F) were obtained from the National Center for Medical Culture Collections (CMCC, Beijing, China). *S.pn* were inoculated on Columbia sheep blood agar plates for 12 to 16 h at 37°C in 5% CO_2_, then transferred into casein hydrolysate plus yeast extract (C+Y) medium at 37°C in 5% CO_2_ until mid-log phase (OD_600_ = 0.4–0.5). Centrifuged at 3500 g for 5 min, the pneumococcal pellet was washed and resuspended to a density of 2×10^8^ CFUs/ml as previously described (18).

### Mouse Model of AOM

The mouse model of AOM was established *via* transbullar injection as described previously (19). In brief, mice were anesthetized with an intraperitoneal injection of ketamine hydrochloride (20 mg/kg of body weight) and xylazine (5 mg/kg of body weight). After exposing bilaterally tympanic bulla, the bony wall of the bulla was fenestrated using a 25-gauge needle and a total of 1×10^6^ CFUs of *S.pn* in about 5 μl was injected slowly into the middle ear cavity with a thinner needle. A mock control cohort of mice was inoculated with an equivalent volume of PBS alone. In some cases, 5 μl containing 2DG (0.2 M) and 1×10^6^ CFU of *S.pn* was injected. After that, the skin incision was sutured. Behavior and weight changes were monitored daily following injection.

### Cell Quantification and Bacterial Load Determination

The tympanic bullae were harvested and spilt into dorsal and ventral halves as described above. The exposed middle ear space was lavaged three times with 40 µl sterile PBS and total middle ear lavage fluid (MELF, about 120 µl) were collected for cell quantification and bacterial load determination. Serial dilutions of the MELF were plated on Columbia sheep blood agar plates for enumeration of pneumococcal load. Another 10 µl MELF was added to 40 µl 1% acetic acid solution for total cell quantification. The rest of MELF were centrifuged at 500 g for 5 min then supernatant of MELF were stored at −80°C until use. The cell pellets were lysed with 700 µl RLT lysis buffer from RNeasy mini kit (QIGEN, USA) after lysed by RBC lysis buffer (Biolegend, USA). After lavaging the middle ear epithelium was harvested with RLT and stored at −80°C.

### Quantitative Real-Time PCR

Total RNA was isolated from middle ear epithelium (MEE) and inflammatory cells in middle ear cavity at designated time points post-infection. Total RNA was extracted by using an RNeasy Minikit according to the manufacturer’s instructions (Qiagen, Valencia, CA). First-strand cDNA was synthesized from 0.5 to 1 µg of total RNA using PrimeScript RT reagent Kit (TAKARA, Japan). qPCR reactions based on SYBR green detection were performed using ABI PRISM 7500 sequence detection system, and quantitative comparisons were obtained using the ΔΔCT method (Applied Biosystems, USA). All reactions were normalized to β-actin. The specific primers for target genes were described in **Table 1**.

**Table 1:** Primer sequences for amplification of mouse gene.

Gene	Primer sequences
glut1	Sense primer: 5′-ATGGATCCCAGCAGCAAG-3′
	Anti-sense primer: 5′-CCAGTGTTATAGCCGAACTGC-3′
hk3	Sense primer: 5′-GGGACACTCTACAAGCTACATC-3′
	Anti-sense primer: 5′-CATCTGGGTAAGGCGACA-3′
pfkfb3	Sense primer: 5′-TTCTCAGGTTTTTGCGGAGAAC-3′
	Anti-sense primer: 5′-GTGCACATGTATGAGCTGGCA-3′
pkm2	Sense primer: 5′-TCGCATGCAGCACCTGATT-3′
	Anti-sense primer: 5′-CCTCGAATAGCTGCAAGTGGTA-3′
ldha	Sense primer: 5′-CAAAGTCCAAGATGGCAACCC-3′
	Anti-sense primer: 5′-AGCACCAACCCCAACAACTGT-3′
mct4	Sense primer: 5′-GCCACCTCAACGCCTGCTA-3′
	Anti-sense primer: 5′-TGTCGGGTACACCCATATCCTTA-3′
HIF-1α	Sense primer: 5′-ACCTTCATCGGAAACTCCAAAG-3′
	Anti-sense primer: 5′-CTGTTAGGCTGGGAAAAGTTAGG-3′
inos	Sense primer: 5′-ACCCTAAGAGTCACCAAAATGGC-3′
	Anti-sense primer: 5′-TTGATCCTCACATACTGTGGACG-3′
β-actin	Sense primer: 5′-ATGGAATCCTGTGGCATCCAT-3′
	Anti-sense primer: 5′-TCCTGCATCCTGTCAGCAATG-3′

### Histology and Immunohistochemistry

Mice were anesthetized and intracardiacally perused with 10 ml 4% paraformaldehyde (PFA) after cardiac perfusing with 10 ml 0.9% saline (NS) at post-challenge. The tympanic bullae were subsequently dissected, fixed overnight in 4% PFA and decalcified for 4 to 6 weeks in 10% EDTA. After that, they were embedded in paraffin, sectioned at 6 µm, stained with hemotoxylin and eosin (HE) and digitally recorded with Nikon ECLIPSE 80i. The thickness of middle ear mucosa was analyzed with NIS-Elements BR 4.10.00 software by two pathologists in a blind fashion. Immunohistochemistry of mouse middle ear tissues were performed to detect expression of HIF-1α. Briefly, slides were dehydrated in xylene and graded alcohols. Antigen retrieval was performed with 0.01 M citrate buffer at pH 6.0 at 95°C for 20 min. the endogenous peroxidase activity was blocked with 0.3% H_2_O_2_ in 0.1 M phosphate-buffered saline (PBS) (pH 7.4). The slides were blocked with PBS containing 1% bovine serum albumin (BSA) or 5% donkey serum, then incubated with primary antibodies to HIF-1α (1:100) at 4°C for overnight. The horseradish peroxidase HRP-conjugated goat anti-rabbit IgG secondary antibodies (Zhongshanjinqiao, Beijing, China) were incubated and developed by the diaminobenzidine substrate kit for peroxidase (Zhongshanjinqiao, Beijing, China). The slides were counterstained with hematoxylin (Zhongshanjinqiao, Beijing, China).

### Immunofluorescence

MELF were pooled from at least three mice. 100 µl MELF were spun onto slides at 500 rpm for 5 min, desiccated for 5 min and then fixed in 4% PFA for 10 min. The slides were blocked with 5% normal donkey serum for 1 h at 37°C and incubated with rabbit anti-pneumococcal polysaccharide polyclonal antibody (1:1000; Staten Serum Institute, Denmark) overnight at 4°C. Secondary antibodies, donkey anti-rabbit IgG conjugated with DyLight 488 (1:1000; Jackson Immuno-Research Laboratories, West Grove, PA) were incubated for 1 h at 37°C. The slides were washed in PBS and counterstained with DAPI (1:800; Invitrogen, USA). Immunofluorescence images were collected using Nikon ECLIPSE 80i.

### Western Blotting

To evaluate the production of HIF-1α protein in middle ear epithelium during AOM, middle ear tissue was pooled from four to five mice and protein were extracted with RIPA buffers. Protein concentration was calculated using bicinchoninic acid (BCA) protein microassay method (Beyotime, China). Samples were loaded on a 10% Tris-tricine gel (Invitrogen Corp), then transferred to polyvinylidene difluoride (PVDF) membranes. The membranes were blocked in 5% BSA including 0.2% Tween 20 TBS and then incubated the primary antibodies to HIF1α (1:2000; #ab16066, Abcam, Cambridge, MA, USA) or β-actin (1: 1,000; Cell Signaling Technology, MA, USA) containing 5% BSA. Blots were washed and incubated with peroxidase-conjugated secondary antibodies (1:10,000; Zhonghshanjinqiao, Beijing, China). Immunoreactive proteins were detected using the ECL (Millipore, Billerica, MA) chemiluminescent system (Amersham Biosciences).

### Total Protein Assay

The amount of total protein in MELF was quantified by bicinchoninic acid (BCA) protein microassay method (Beyotime, China) according to the manufacturer’s instructions. Absorbance was read at 562 nm.

### Activity Assay

The level of glucose and lactate in supernatant of MELF were determined by using the glucose assay kit (Applygen, Beijing, #E1011) the lactate assay kit (St. Louis, MO, USA, #MAK064) according to the manufacturer’s instructions, respectively. The lactate dehydrogenase (LDH) in supernatant of MELF released by damaged cells was measured by LDH assay kit (Beyotime, China, A020-1) according to the instructions.

### Cytokine Measurements

Commercially available enzyme linked immunosorbant assay (ELISA) kits were used to measure the concentrations of IL-1β, TNF-α, IL-6 (Biolegend, San Diego, USA), CXCL1, and CCL2(R&D Systems, Minneapolis, MN, USA) according to the manufacturer’s instructions.

### Flow Cytometry

The MELF of 5 mice from each group at 1 day and 3 days after *S.pn* inoculation were pooled, centrifuged at 500 g. The cell pellets were lysed with RBC lysis buffer (Biolegend, USA) and washed twice with FACS buffer. Fc receptors were blocked with Mouse BD Fc Block (BD Biosciences, San Diego, CA) and cells were stained with specific immune cell surface markers. The following staining parameters were employed: neutrophils as CD11b+Ly-6G+, Macrophages as CD11b+F4/80+ and their isotype antibodies were added for 45 min on the ice. Lastly, all samples were washed three times and resuspended in FACS stain buffer prior to analysis on a BD FACS Calibur flow cytometer (BD Biosciences, USA).

### Phagocytosis Assays

FITC-labeled *S.pn* were prepared by incubation with 4 mg/ml FITC (Sigma, Poole, UK) for 30 min at 4°C. Peritoneal neutrophils (5 ×10^5^ cells) were incubated with FITC-labeled bacteria (at multiplicity of infection, MOI, of 100) for 30 min at 37°C. After washing steps, cell nuclei were stained with DAPI (Invitrogen), followed by visualization using confocal laser scanning microscopy (LSM 510, Zeiss). The ratio of engulfed bacteria (as determined by overlay of green bacteria) was quantified by an independent researcher from 100 counted cells per well and was expressed as percentage of cells that contain bacteria.

### Bacterial Killing Assays

*S.pn* were grown to logarithmic phase in C+Y to OD _600_ = 1×10^8^ CFUs/ml. Peritoneal Neutrophils were infected with *S.pn* (at multiplicity of infection, 100) for 0.5 h for evaluation of bacterial uptake, then immediately, extracellular bacteria were removed by washing with 10 µg/ml Penicillin and 200 µg/ml gentamicin. Neutrophils were continued to incubate 1 h for evaluation of intracellular killing (total time=1.5 h), respectively. Cells were lysed and live intracellular bacteria loads were determined by plating cell lysate on Columbia CNA agar plate. The colonies were counted after overnight incubation at 37°C in a 5% CO_2_ atmosphere. Intracellular killing was calculated as follows: (number of CFUs_t=0.5 h_ − number of CFUs _t=1.5 h_)/number of CFUs_t = 0.5 h_.

### Statistical Analyses

Data are presented as the mean ± standard deviation (SD) of 3 independent experiments. All statistical analysis was performed using GraphPad prism software version 5.01 for Windows (GraphPad, USA). Independent t tests were used for data with normal distribution. The statistical significance was set at *p* < 0.05.

## Results

### Glycolysis Increases in Response to *S.pn* infection During AOM

**Figure 1:**
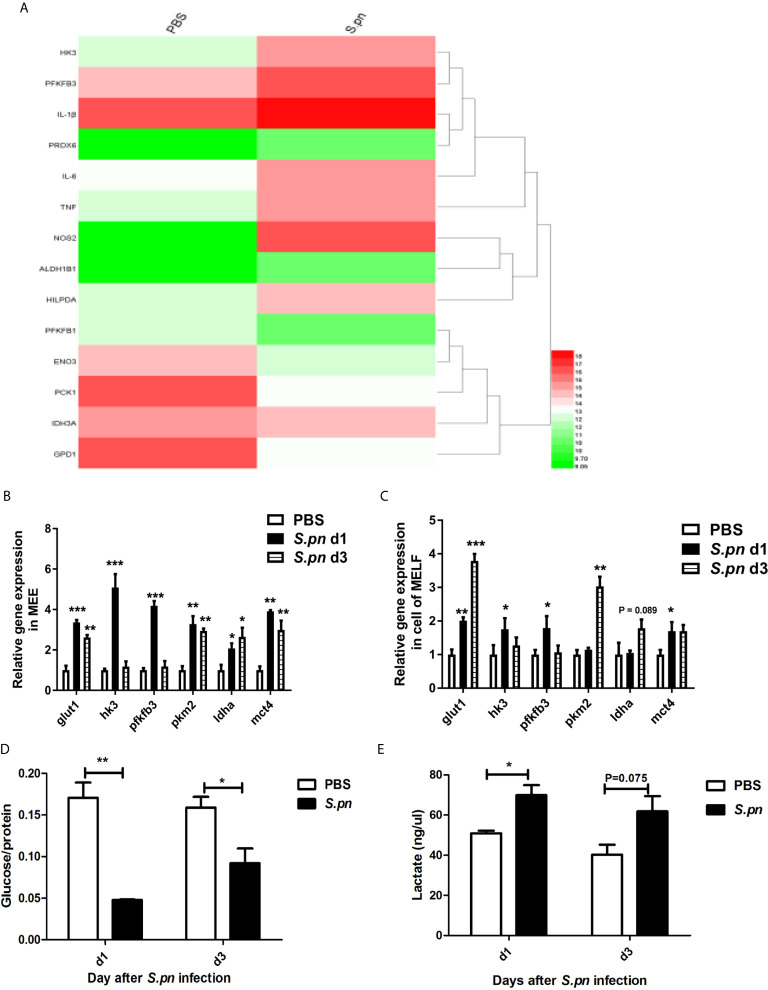
Glycolysis increases in response to S.pn infection during AOM. **(A)** The expression of glycolysis-related genes in mouse middle ear was detected by microarray. Red to green on the heat map indicated the expression level was gradually downregulated. **(B)** The mRNA expression levels of glycolysis-related genes in middle ear epithelium and **(C)**, middle ear cavity inflammatory cells were verified by RT-qPCR at designated time points post-infection. **(D)** Levels of glucose and **(E)**, lactate in the supernatants of MELF were detected refer to the manual. Data represent three independent experiments and are presented as mean ± SD (n = 3). *P < 05; **P <.01; ***P < 001.

To get the whole mRNA expression profiles of metabolic changes in *in vivo* experiment, microarray was used to screen differentially expressed genes (DEGs) in mouse middle ear. Among the 952 DEGs, hexokinase 3 (hk3), fructose-2,6-biphosphatase3 (pfkfb3) and inducible nitric oxide synthase 2 (inos2) were significantly up-regulated, while glycerol-3-phosphate dehydrogenase 1 (gpd1) and phosphoenolpyruvate carboxykinase1 (pck1) were significantly down-regulated, suggesting increased glycolysis in response to *S.pn* infection during AOM (**Figure 1A**). Next, the expression levels of glycolysis-related genes were verified by real-time PCR at day 1 and 3 post-infection, indicating an obvious increase of key glycolytic enzymes and proteins in MEE (**Figure 1B**) and inflammatory cells of MELF (**Figure 1C**), including hk3, pfkfb3, pyruvate kinase M2 (pkm2), lactate dehydrogenase a (ldha), glucose uptake transporter 1 (glut1) and lactate secretion transporter 4 (mct4). Furthermore, compared with PBS control, the glucose and lactate concentration were measured to have significantly decreased and increased respectively (**Figures 1D, E** **)**, supporting that glycolysis increased in response to *S.pn* infection during AOM.

### Glycolysis Promotes Inflammatory Responses During AOM

To assess the effect of glycolysis on the inflammatory response, 2DG was used to inhibit glycolysis. First, trypan blue rejection assay was used to confirm that 2DG produce no cytotoxicity to neutrophils *in vitro* (data not shown). Next, compared with *S.pn* alone group, the expression levels of glycolysis-related genes, such as hk3, pfkfb3 and ldha, decreased remarkably in MEE in *S.pn*+2DG treatment group, while glut1, pkm2, and mct4 displayed a tendency to decline (**Figure S1A**). Meanwhile, the expression level of mct4 decreased obviously in inflammatory cells of MELF in *S.pn* +2DG treatment group, while glut1, hk3, pfkfb3, pkm2, and ldha had a tendency to decline (**Figure S1B**). Furthermore, the levels of glucose and lactate in supernatant of MELF were measured. Compared with *S.pn* alone group, the glucose concentration significantly increased (**Figure S1C**) and the lactate concentration significantly decreased (**Figure S1D**) in *S.pn* +2DG treatment group, indicating the inhibition efficacies of 2DG on glycolysis.

**Figure 2:**
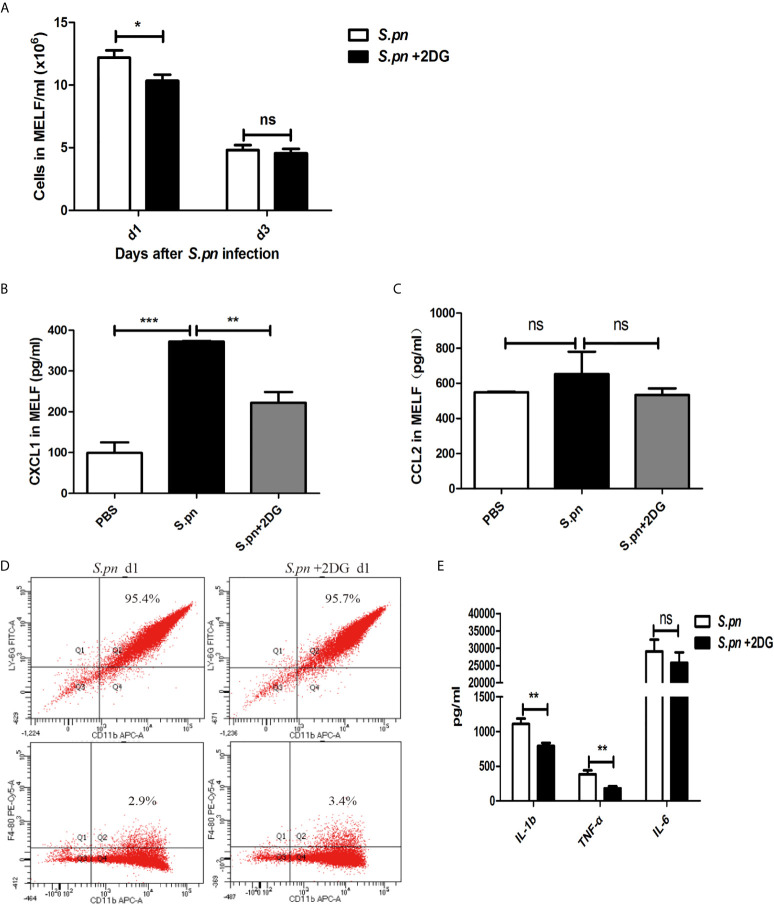
Glycolysis promotes inflammatory responses during AOM. **(A)** Inflammatory cells in MELF at designated time points post-infection were quantified (n=18). **(B)** Chemokines CXCL1 and **(C)**, CCL2 in MELF were detected by ELISA. **(D)** The proportion of neutrophils (CD11b+Ly6G+) and macrophages (CD11b+F4/80+) in the MELF was detected by flow cytometry. **(E)** Cytokines in MELF were detected by ELISA. Data represent three independent experiments and are presented as mean ± SD (n = 3). *P <.05; **P <.01; ***P <.001. ns, not significant.

Next, we observed a notable decrease of the number of inflammatory cells (**Figure 2A**) and the level of chemokine CXCL1 (**Figure 2B**) in *S.pn*+2DG treatment group compared with *S.pn* alone group, along with a down-regulation trend of CCL2 level (**Figure 2C**), suggesting that glycolysis inhibitor 2DG could reduce the recruitment of inflammatory cells by down-regulating the expression of chemokines. Furthermore, the inflammatory cells of MELF were classified and quantified by flow cytometry. As **Figure 2D** showed, more than 95% of the MELF cells were neutrophils (CD11b+Ly-6G+), and only about 2% to 3% were macrophages (CD11b+F4/80+). In addition, the proportion of cell types in MELF was not altered by 2DG treatment. Meanwhile, the levels of pro-inflammatory cytokines in MELF were detected by ELISA. Compared with *S.pn* infection alone group, the levels of IL-1β and TNF-α significantly decreased in *S.pn*+2DG treatment group, but there was no significant difference in the level of IL-6 (**Figure 2E**). Above results indicated that glycolysis promotes the recruitment of inflammatory cells and the production of IL-1β and TNF-α during AOM.

### Glycolysis Contributes to Bacterial Clearance by Enhancing Phagocytosis and Killing Capability of Neutrophils

**Figure 3:**
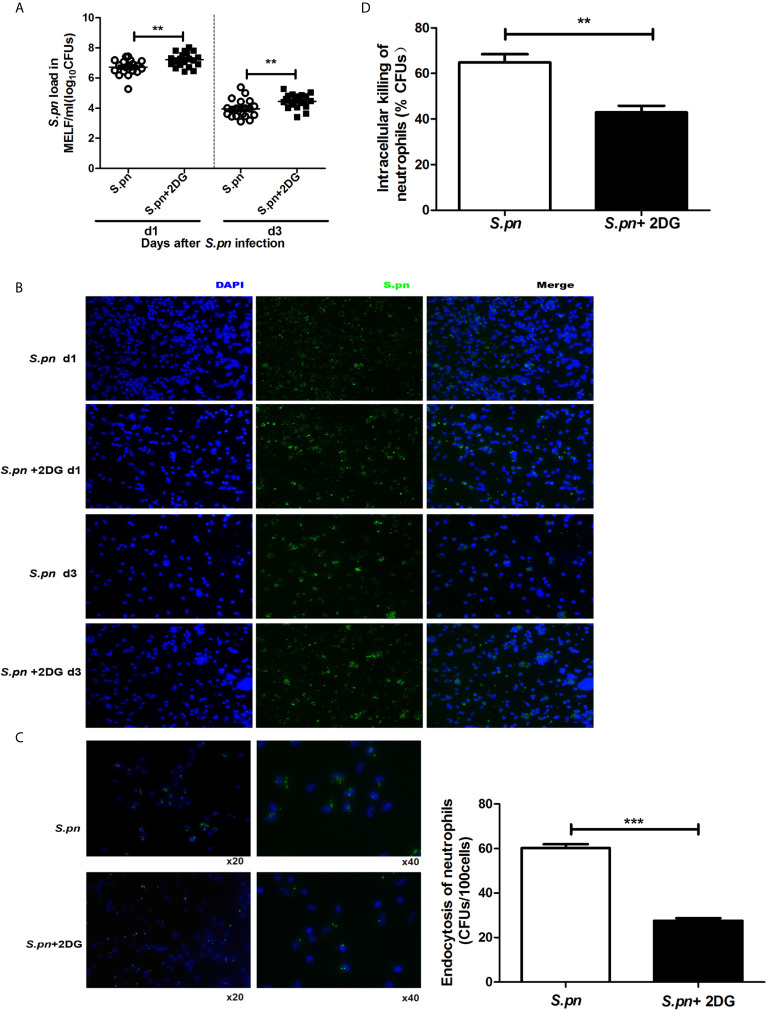
Glycolytic pathway facilitates the clearance of *S.pn*. **(A)** *S.pn.* burden in MELF was counted after *S.pn.* ± 2DG treatment (n=18). **(B)** *S.pn.* loads in MELF were shown by immunofluorescence after *S.pn.* ± 2DG treatment. **(C)** Number of FITC-labeled *S.pn.* phagocytosed by 100 neutrophils. **(D)** The intracellular killing ratio of neutrophils was shown. Data represent three independent experiments and are presented as mean ± SD (n = 3). **P <.01; ***P <.001.

Since glycolysis has been shown to promote bacterial clearance, the effect of glycolysis on phagocytosis and killing capability of neutrophils was investigated. It was worth noting that the *S.pn* load in *S.pn*+2DG group became significantly higher than *S.pn* infection alone group on day 1, 3 (**Figure 3A**). Additionally, immunofluorescence staining of the MELF cytospin specimens showed that a large amount of intact pneumococci were detected in *S.pn*+2DG group on day 1, 3, in contrast to a small amount of pneumococcal debris in *S.pn* alone group (**Figure 3B**). Further, we conducted *in vitro* experiments to examine the phagocytosis and killing capability of neutrophils, which are the dominating cells during the phase of AOM inflammation response. As expected, the phagocytosis and killing capability of neutrophils were significantly suppressed (**Figures 3C, D** **)** after 2DG treatment, suggesting a positive role of glycolysis to bacterial clearance by enhancing phagocytosis and killing capability of neutrophils.

### Glycolysis Aggravates Middle Ear Tissue Injury

**Figure 4:**
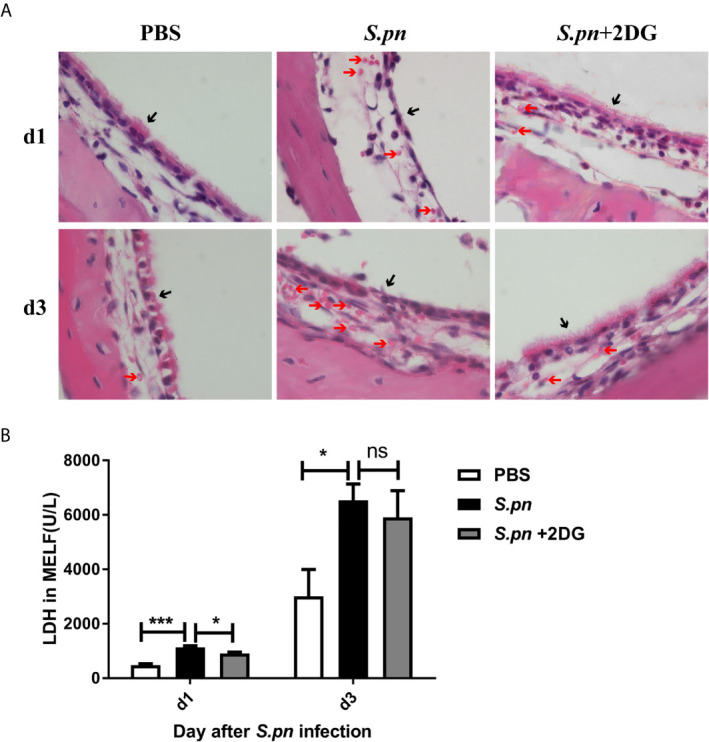
Glycolysis aggravates middle ear tissue injury. **(A)** Middle ear epithelial damage was assessed by HE staining. **(B)** LDH activity in the supernatants of MELF was shown. Data represent three independent experiments and are presented as mean ± SD (n = 3). *P <.05; ***P <.001. ns, not significant.

Since inflammatory response is generally viewed as an effector causing local tissue damage, we hypothesize that increased glycolysis not only triggers a robust inflammatory response, but also leads to middle ear injury during AOM. Comparing the pathological changes of ME mucosa near the eustachian tube orifice, we observed the ameliorative effect on the mucosal thickness, epithelium integrity and red cell infiltration in *S.pn*+2DG treatment mice than in *S.pn* infection alone mice (**Figure 4A**). Moreover, the level of LDH, an injury marker, was seen to undergo a dramatic decrease in *S.pn*+2DG treatment group compared with *S.pn* infection alone group on day 1 post-infection, with a decrease trend on day 3 (**Figure 4B**). Taken together, our data demonstrated that enhancement of glycolysis induced by *S.pn* aggravates middle ear tissue injury during AOM.

### HIF-1α Participates in Glycolysis Conversion During AOM

**Figure 5:**
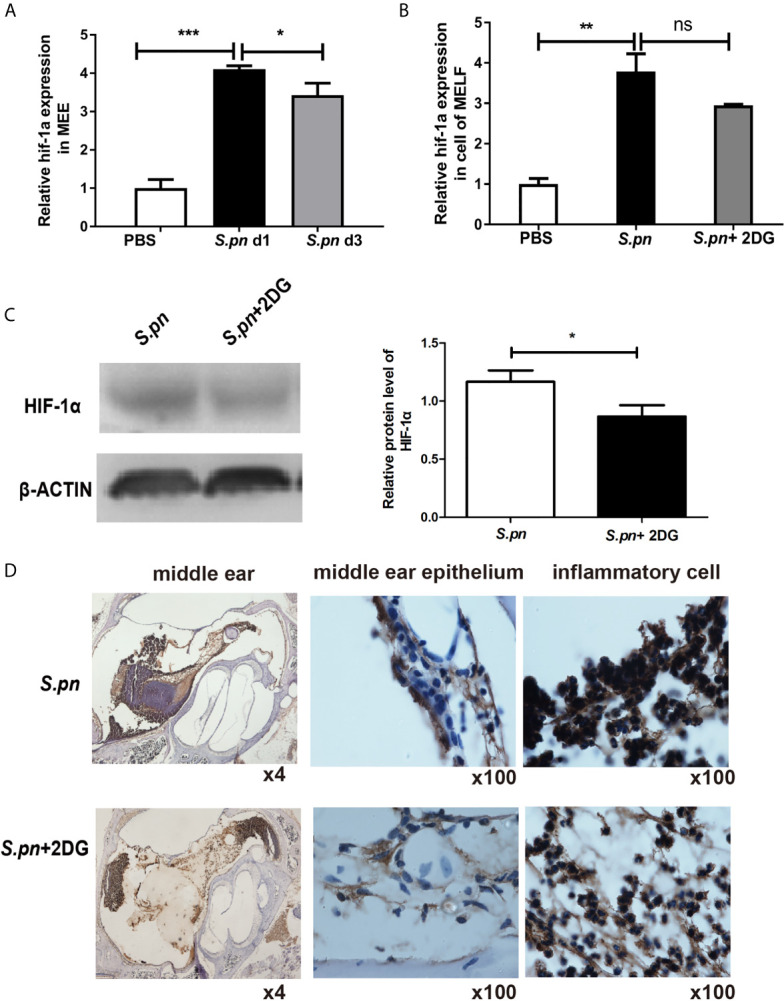
HIF-1α participates in glycolysis induced by *S.pn* infection during AOM. **(A)** HIF-1α mRNA expression in MEE and **(B)**, inflammatory cells number in MEC was detected by RT-qPCR. **(C)** Protein level of HIF-1α in MEE was detected by western blot assay after *S.pn* ± 2DG treatment at days 1 post-inoculation. **(D)**Protein level of HIF-1α in MEE and inflammatory cells was detected by IHC. Data represent three independent experiments and are presented as mean ± SD (n = 3). *P <.05; **P <.01; ***P <.001. ns, not significant.

To verify whether HIF-1α participates in metabolic changes and inflammatory defense during AOM, the expression levels of HIF-1α were measured by qRT-PCR and IHC. As shown in **Figures 5A, B**, the mRNA expression of HIF-1α was significantly up-regulated both in MEE and cells of MELF at day 1 post-infection compared to PBS control, consistent with the expression levels of glycolysis-related genes. Immunohistochemistry staining also demonstrated the same results (**Figure S2**). To further investigate the effect of glycolysis on HIF-1α expression, 2DG was used to inhibit glycolytic metabolism. As expected, reduced expression of HIF-1α mRNA in MEE (**Figure 5A**) and cells of MELF (**Figure 5B**) was observed in S.pn+2DG treatment group. Immunohistochemistry and Western blot also demonstrated the levels of HIF-1α protein decreased after 2DG treatment (**Figures 5C, D** **)**, suggesting that HIF-1α participates to glycolysis during S.pn AOM.

## Discussion

The immune system encompasses a heterogeneous population of cells that for the most part are relatively quiescent in the steady state, but share the ability to rapidly activate and then generate immune responses upon infection, inflammation, and other stimulants (33). A large amount of energy is needed in the process of cell activation and immune response from relatively static state, among which ATP is the most direct energy supplier. Intracellular ATP is produced mainly through the decomposition of glucose. The glucose catabolism pathways of ATP production include anaerobic oxidation and aerobic oxidation, during which glycolysis is the common initiation pathway of both. In addition, there is a general view that myeloid cells primarily use glycolysis as a source of ATP (31). Therefore, we focused our interest on how middle ear epithelial cells and immune cells regulate metabolic pathways to support or guide functional changes during *S.pn* AOM.

Glycolytic pathway requires the involvement of multiple enzymes and transporters, such as GLUT1, HK3, PFKFB3, PKM2, LDHA, MCT4, etc. In particular, GLUT1 is the transporter molecule that initiates glycolysis by transferring glucose into cells. HK3 is a key enzyme catalyzing the generation of glucose 6-phosphate in the first step of glycolysis. PFKFB3 promotes glycolysis as a strong allosteric activator of 6-phosphofructokinase 1 (PFK1). PKM2, as the allosteric enzyme of pyruvate kinase M (PKM), catalyzes phosphoenolpyruvate to pyruvate. LDHA catalyzes pyruvate to lactic acid, and MCT transports the lactic acid from inside to outside of the cell. In our study, the analysis of gene chip revealed an increase of glycolysis-related genes expression in middle ear epithelium (MEE) during *S.pn* induced AOM. Consistent with the results of microarray, qPCR confirmed that the levels of glycolysis-related genes, such as glut1、hk3、pfkfb3、pkm2、ldha and mct4, increased in MEE and cells of MELF. In addition, spectrophotometry detected a glucose decrease and lactate increase, verifying the level changes of glycolysis in the supernatant of MELF during AOM. Above results suggested that middle ear epithelial cells and immune cells might upregulate glycolysis metabolic pathways to support or guide functional changes during AOM.

Accumulating evidence showed that glycolysis not only regulates cell migration and exudation, but also plays an important role in cellular immune function. During the process of energy metabolism, the upregulation of key glycolytic genes and proteases and the accumulation of intermediate metabolites in immune cells affects the function of immune cells, including phagocytosis and killing of pathogens, release of antimicrobial substances and pro-inflammatory cytokines (12–17). A recent study has shown that mycobacterium tuberculosis infection promotes glycolysis in human alveolar macrophages (30, 34). After inhibition of glycolysis, the level of IL-1β decreased and bacterial load increased, suggesting that glycolysis can inhibit bacterial growth by promoting the release of pro-inflammatory cytokines (17, 35). Similarly, our results showed that the bacterial load in the MEC increased significantly after glycolysis inhibitor 2DG treatment, while pro-inflammatory cytokines TNF-α and IL-1β significantly downregulated, indicating that glycolysis may promote *S.pn* clearance by enhancing the middle ear inflammatory response. Our previous studies showed that neutrophils are the main cells (>98%) involved in the clearance of *S.pn* during AOM (18), and neutrophils rely mainly on glucose *via* aerobic glycolysis to support ATP production and maintain bactericidal function (15, 36). Accordingly, we evaluated the role of glycolysis in the phagocytosis and killing function of neutrophils in *in vitro* experiments. As expected, the phagocytosis and killing capability of neutrophils were significantly suppressed by 2DG treatment, indicating that glycolysis could also promote bacterial clearance by enhancing the phagocytosis and killing ability of neutrophils.

It has been shown that tissue damage is usually not directly caused by pathogenic infections, more often than not, it is caused by the host’s excessive immune response. Numerous studies have shown that glycolysis plays an important role in cellular immune function. In agreement with the notion, our research confirmed that 2DG-inhibited glycolysis can reduce the middle ear tissue damage by limiting the inflammatory response. Above studies showed that metabolic conversion could regulate the host defenses and prognosis of inflammatory responses, making it possible to develop a potential novel target for controlling excessive inflammation and improper immune responses.

HIF-1α is a key regulator of glycolysis that promotes the interaction of intracellular PKM2 with HIF-1α *via* the NF-κB pathway, which regulates transcription of early pro-inflammatory cytokines (such as TNF-α and IL-1β, etc.) and other related genes (such as GLUT1, LDHA, PDK1, etc.) (9, 37). Study has found that glycolytic rate and metabolic capacity were significantly reduced in HIF-1α-defective macrophages, as well as reduced cellular migration (7, 16). Our study also found a robust increase of mRNA levels and protein levels of HIF-1αin the middle ear epithelial cells and exudative inflammatory cells after *S.pn* infection. Unfortunately, we have not yet elucidated the molecular mechanism of HIF-1α on regulating glycolysis and immune responses in the course of AOM.

In conclusion, increased glycolysis conversion, induced by *S.pn* infection in the middle ear of AOM mice, promotes the recruitment of inflammatory cells and the production of inflammatory factors, contributes to bacterial clearance, but also aggravates tissue damage. This study suggests that metabolic changes can regulate host defense and the prognosis of inflammatory responses, shedding light on the potential of immunometabolism as a novel therapeutic target for AOM.

## Data Availability Statement

The raw data supporting the conclusions of this article will be made available by the authors, without undue reservation.

## Ethics Statement

The animal study was reviewed and approved by the Institutional Animal Care and Use Committee at Chongqing Medical University.

## Author Contributions

FF, YM, and YH conceived of and designed the research. FF, YM, RA, ZD, DL, YZ, QH, XZ, and YD performed the experiments and analyzed the data. FF, YM, and YH wrote the manuscript. ZD and YH interpreted the data and corrected the manuscript. All authors contributed to the article and approved the submitted version.

## Funding

This study is supported by National Natural Science Foundation grants of China (81373151) and Natural Science Foundation Project of CQCSTC (cstc2018jcyjAX0257).

## Conflict of Interest

The authors declare that the research was conducted in the absence of any commercial or financial relationships that could be construed as a potential conflict of interest.

## Supplementary Material

Supplementary Figure 1 Glycolytic inhibitor 2DG inhibits glycolytic metabolism in the middle ear during AOM. **(A) **mRNA expression levels of glycolysis-related genes in MEE and **(B)**, inflammatory cells in MEC, assessed by RT-qPCR. **(C)** Levels of glucose and **(D)**, lactate in the supernatants of MELF were detected refer to the manual. Data presented as mean ± SD (n = 3). *P <.05; **P <.01; ***P <.001.

Supplementary Figure 2 Protein expression level of HIF-1α on middle-ear and inflammatory cells number in MEC were detected by immunohistochemical at days 1,3,5,7 after PBS or S.pn treatment.
