# Supplementary figures and images for: Glycolytic Metabolism Is Critical for the Innate Antibacterial Defense in Acute Streptococcus pneumoniae Otitis Media
Source: Front Immunol. 2021 Apr 19;12:624775. doi: 10.3389/fimmu.2021.624775 (PMC8089373; doi:10.3389/fimmu.2021.624775)

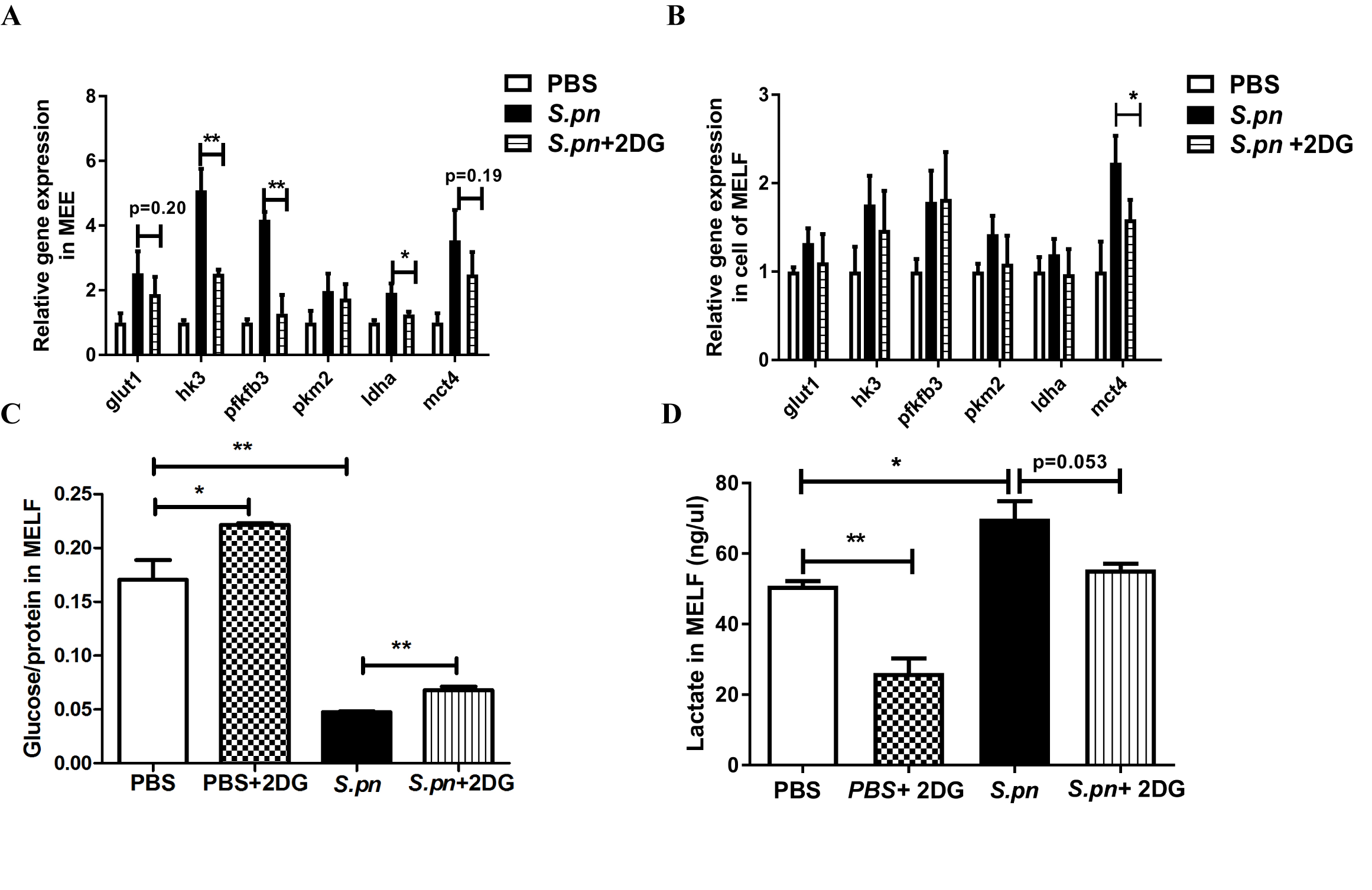

Supplement: Supplementary Figure 1 — Glycolytic inhibitor 2DG inhibits glycolytic metabolism in the middle ear during AOM. (A) mRNA expression levels of glycolysis-related genes in MEE and (B), inflammatory cells in MEC, assessed by RT-qPCR. (C) Levels of glucose and (D), lactate in the supernatants of MELF were detected refer to the manual. Data presented as mean ± SD (n = 3). *P <.05; **P <.01; ***P <.001. [file Image_1.tif]

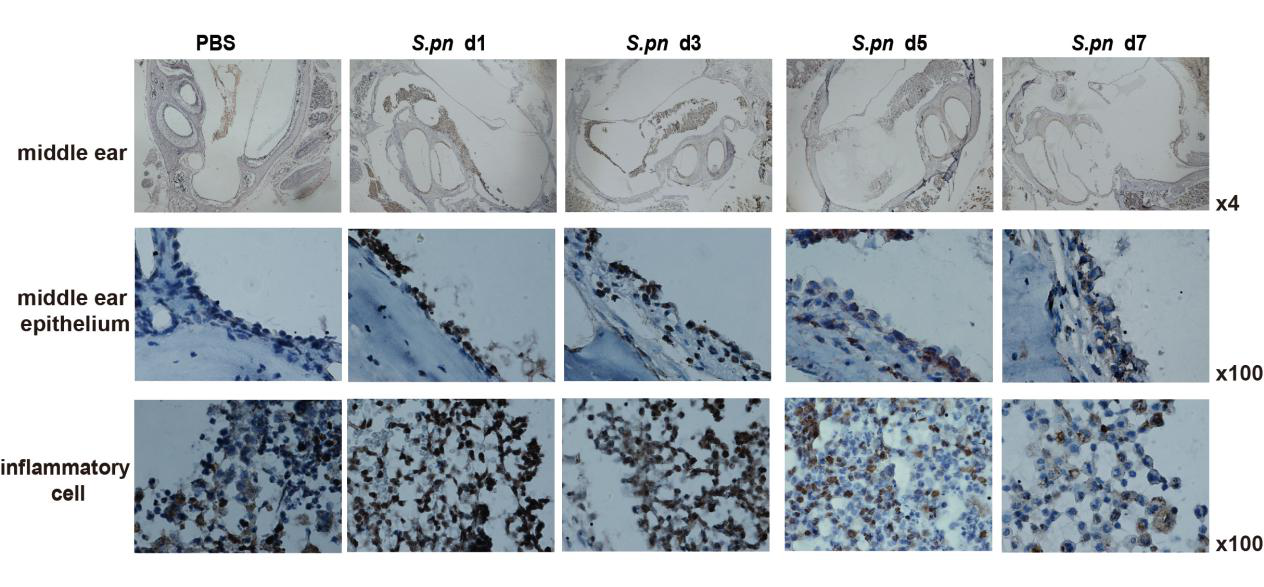

Supplement: Supplementary Figure 2 — Protein expression level of HIF-1α on middle-ear and inflammatory cells number in MEC were detected by immunohistochemical at days 1,3,5,7 after PBS or S.pn treatment. [file Image_2.tif]
